# Risk factors of cervical cancer and role of primary healthcare providers regarding PAP smears counseling: Case control study

**DOI:** 10.12669/pjms.38.4.4969

**Published:** 2022

**Authors:** Halima Sadia, Irfan Murtaza Shahwani, Kiran Fatima Mehboob BANA

**Affiliations:** 1 Dr. Halima Sadia, MBBS. Resident, Department of Family Medicine, Ziauddin University, Karachi, Pakistan; 2 Dr. Irfan Murtaza Shahwani, MBBS. Resident, Department of Family Medicine, Ziauddin University, Karachi, Pakistan; 3 Dr. Kiran Fatima Mehboob BANA, BDS, MCPS-HCSM, MHPE. Assistant Professor in Department of Medical Education, Bahawalpur Medical & Dental College, Bahawalpur, Pakistan

**Keywords:** Cervical cancer, Counseling, PAP smear, Primary healthcare providers

## Abstract

**Objectives:**

To determine risk factors of cervical cancer and role of healthcare providers regarding awareness and counseling of PAP-smear.

**Methods:**

It was case-control study conducted from Jan-2021 till may-2021 at two tertiary care hospitals of Karachi South. The intended sample size was 255 subjects as 105 cases and 150 controls. The inclusion criteria were diagnosed cases of CA Cervix and under the age of 50 years. Controls were recruited from the general population. All the data were entered into SPSS version 23. The odds ratio was calculated to compare the risks of occurring CA cervix among cases and controls. The relationship of risk factors was assessed by binary logistic regression. P-value < 0.05 was considered as statistically significant.

**Results:**

Generally, highly significant p-value (<0.000) was observed; depicted a positive association for a level of education, age at first intercourse, and number of parity in cases of CA cervix and controls (OR=4.3). The correct predicted rate was 68.8% for having CA cervix among controls due to family history, the knowledge of PAP smear screening, ever counseled for a PAP smear, ever tested for a PAP smear, never been tested for PAP smear due to cost.

**Conclusion:**

Educational level, age at first intercourse, number of parity was the risk factors of CA cervix. Family history of CA Cervix, knowledge of cervical cancer screening, ever counseled for PAP smear, ever tested for PAP smear, never been tested for PAP smear due to cost were significantly predicted for CA cervix among controls.

## INTRODUCTION

Cervical cancer is amongst the top ten common cancers worldwide and reported as third commonly occurring 5.98% malignancy among female in Pakistan.^1^ Globally nearly 528,000 women per year are diagnosed with this aggressive carcinoma of cervix in 2012 and 99.7% cancers were caused by high risk human PAPilloma virus.^2^ In Pakistan, according to International Agency for research on Cancer (IARC) and HPV information center revealed that in 2018 nearly 5,601 incidences of cervical cancer were reported from which 3,861 were deceased.^3^ Cervical cancer is a developing and killing cancer among all ages of Pakistani women.^4^ Despite preventable nature of cervical cancer; ignorance towards screening, discomfort, shame, fear, embarrassment, fallacies regarding screening, vaccination and screening cost placed a huge burden on healthcare system of low and middle income countries such as Pakistan.^5^ Therefore; mortality rate is high in cervical carcinoma as more than 70% females reported to physicians in advance stage of disease.^4^

The identified risk factors of cervical cancer includes unprotected sex, poly gammy, poor socioeconomic status, early marriages, low education level, early reproductive cycle, multi parity, smoking, co-infections, HPV infections, altered hormones and immune system.^2^ Sexually transmitted infection such as high risk human papilloma virus (HPV) type 16, 18, and 31 are oncogenic in nature and cause cervical cancer.^6^,^7^ Cervical cancer originates from HPV infection and progresses slowly with time. HPV infection sometimes converts into premalignant lesion and later transform into advance invasive nature if not attained on time. As reported by Qureshi MA et al^8^; the incidence of cervical carcinoma in women of (Karachi) Pakistan according to Dow registry is low as 1.3% as compare to developed countries which is 13.1% but have high mortality rate due to delayed reporting when cancer becomes aggressive. Therefore, the age endorsed by World Health Organization for regular screening of cervical cancer through PAP smear is 30-49 years. The screening is recommended from 50-65 years with the interval of three years.^5^

PAP (PAPanicolaou) smear is a gold standard screening test among asymptomatic females and can drop 2.6% an average mortality cases from cervical cancer per year in vigorous health care systems.^5^,^9^ PAP smear is one of the effective screening tools to detect cervical cancers on early stage. The sensitivity of PAP smear is 70% to detect aggressive tumor of squamous intraepithelial lesion.^5^,^10^ DNA testing has also high sensitivity in association with HPV to detect premalignant lesions promptly.^9^ It is well known that prevention is better than cure. However, various cost effective and evidence based innovative approaches have been proven to prevent cervical cancer such as “screen and treat” included HPV vaccination and HPV testing.^6^ There are various studies conducted worldwide to combat the burden of cervical cancer through awareness and education campaign.^7^,^11^ In this regard, impact of education on healthy behavior and lifestyle modification has been studied to prevent human PAPilloma virus infection and cervical carcinoma.^7^,^11^

Various reviews have been executed at national level to determine the knowledge of cervical cancer and frequency of PAP smear testing in local population.^4^,^5^,^12^ There is dearth of literature available regarding the role of primary health care provider about PAP smear counseling and risk factors of cervical cancer and indeed it was the rationale of the study. Therefore; this study was aimed to determine the risk factors of cervical cancers and role of healthcare providers regarding awareness and counseling of PAP-smear among females of Karachi South.

## METHODS

It was a case control study design conducted from Jan-2021 till may-2021 at two tertiary care hospitals of Karachi South. This study was approved by institutional ethical review board of BUMDC numbered 59/2021received on jan-2021. The sample size was calculated by the help of standard formula on the basis of 3% awareness of cervical cancer screening in Punjab Pakistan^13^ as 45. Hence the intended sample size was 255 subjects as 105 cases and 150 controls. The proportion of cases verses controls was 1:2. Through consecutive sampling technique; the cases of CA Cervix were selected. The inclusion criteria of cases were female with the diagnosed case of CA Cervix and under the age of 50 years. Controls were recruited by frequency matching of age and socio-economic status from general population. The written consent was obtained by the participants and rationale of the study was well explained to the subjects before data collection. Anonymity of data was well assured to the participants. Risk factors of cervical cancer and role of primary healthcare providers regarding PAP smear counseling was assessed by using the structured questionnaire in both groups. The questionnaire was formulated by evidence based extensive literature search^10^,^11^,^13^ and then validated by five healthcare providers having additional qualification in medical education. The corrections were incorporated later in the final questionnaire as modification of some items. The reliability was checked by pilot testing among 15 females for proper comprehension in Urdu language. The questionnaire have three sections; the first section was regarding the demographic variables and reproductive characteristics such as age, education level, living area (urban/rural), age at first intercourse, number of parity, first marriage of both couple, multiple sex partners and medical history; the second section addressing the knowledge of PAP smear, ever been tested for PAP smear and cost of PAP-smear test, the third section was regarding the influence of awareness and counseling among cases and controls. The responses were recorded as Yes or No. All the data were entered into SPSS version 23. Normality of data was checked by Shapiro Wilk test. The odds ratio was calculated to compare the risks of occurring CA cervix amid controlled group. The relationship of risk factors in cases and controls was assessed with binary logistic regression. P-value < 0.05 was considered as statistically significant.

## RESULTS

Total 255 questionnaires were filled from which 250 were completed in all aspects therefore; response rate was 98%. There were total n=100 cases and n=150 controls. Majority of participants were from 40-49 years as 47% and 53% from cases and controls respectively. Total 29% cases and 8.6% controls were got married at an early age of 11-18 years. Total 23% and 10.6% women were uneducated amid cases and controls respectively. Hence age, age at first intercourse and educational status were significantly associated variables at p-value=0.000 in both groups-Table-1.

**Table-I:** Association of demographic Variables in Cases and Controls.

Demographic Variables		Cases n=100	Controls n=150	P-Value*
Age	30-39 Years	31	61	0.000
	40-49 Years	47	80	
	50-59 Years	22	9	
Living Area	Urban	54	71	0.183
	Rural	46	79	
Educational Status	Primary	16	7	0.000
	Secondary	42	83	
	Graduation	19	44	
	Uneducated	23	16	
Age at first intercourse	11-18 Years	29	13	0.000
	19-25 Years	53	91	
	>26 Years	18	46	

* Chi-Square.

Univariate analysis was performed to estimate the risk factors amid cases and controls. Generally, highly significant p-value (<0.000) was observed; depicted positive association for age at first intercourse at (OR=4.3). In addition; positive association with OR of 2.67 with statistically significant p-value =0.001 for first marriage of both couples and 6% and 11% for multiple sex partners amid cases and controls respectively as a risk factor Table-II.

**Table-II:** Univariant Analysis of Risk factors of Cervical Cancer among Cases and Controls.

Variables		Cases n=100	Controls n=150	Odds Ratio	CI	P-Value
Educated	Yes	77	134	0.4	0.2-0.8	0.000
	No	23	16			
Age at first intercourse	11-18 Years	29	13	4.3	2.11-8.79	0.000
	19-25 Years	53	91			
	>26 Years	18	46			
Number of Parity?	0	16	14	0.55	0.31-0.97	0.062
	1-3	61	83			
	>4	23	53			
Is this first marriage of both couples?	Yes	83	97	2.67	1.44-4.96	0.001
	No	17	53			
Multiple sexual partners	Yes	6	17	0.5	0.19-1.31	0.112
	No	94	133			

Variable(s) entered on step 1: Family history of CA Cervix, importance of PAP smear, ever counseled for PAP smear, ever tested for PAP smear, never been tested for PAP smear due to cost

The binary logistic regression test was performed to assess the influence of family history of CA cervix, importance of PAP smear, ever counseled for PAP smear, ever tested for PAP smear, never been tested for PAP smear due to cost among the participants. The results demonstrated statistically significant improvement in constant predictor model, X^2^ (5, N=250) =43.037, p=0.000. The Wald test depicted that family history of CA Cervix, importance of PAP smear, ever counseled for PAP smear, ever tested for PAP smear, never been tested for PAP smear due to cost were significantly predicted for CA cervix among controls. The Nagelkerke R^2^ showed 21% total variance in model. The correct predicted rate was 68.8% for having CA cervix among controls due to lack of awareness and counseling of PAP smear.

## DISCUSSION

This study was aimed to determine the risk factors of cervical cancer and role of healthcare providers regarding awareness and counseling of PAP-smear among cases and controls. It was evident from the literature that low education level,^14^ early marriages, low socio-economic status ,multi parity, un protected sex, poly gammy and co-infections are the risk factors leads to carcinoma of cervix.^6^,^9^ Due to lack of awareness, the cases are reported to health care providers when it transforms into aggressive stages and hence leads to mortality. The survival rate is higher when cases are reported on early stage and on the contrary there is 100% mortality rate after five years when cases are diagnosed at stage IV as reported by Baeten et al.^15^

The result of this study revealed that certain behavioral and demographic covariates such as level of education, age at first intercourse, multi parity and first marriage of both couples have detrimental effects on cervical health among cases and controls are more prone to have CA cervix due to influence of these risk factors. The results of our study are in accordance with the study of Jehan et al.^16^ This study reported the evaluation of risk factors among 103 young females at Aga Khan Hospital Karachi (AKUH) and revealed that early marriages were proved to be a strong risk factor as chances of abnormality is reduced over 26 years. Young females are at more risk of developing cervical cancer of aggressive stage then before in recent data from Pakistan.^17^

Cervical cancer is treatable and preventable agony; if diagnosed at an early stage. CA cervix can be prevented by two preventive modes; primary prevention is to vaccinate young women before marriage as proposed by WHO and secondary prevention is screening test.^16^ Gerdasil and Cervarix are two globally recommended vaccines against HPV.^16^ The regular PAP smear screening test with the interval of three years, limited sex partners, quit smoking, vaccination against HPV and protected sex are the ways to prevent CA cervix.^18^

PAP smear screening is reliable and easy test to identify cervical cancer in early stage. As a result of mass awareness and screening campaigns in developed countries; the incidence of cervical cancer has declined in past 30 years but in developing countries; the cases are intensifying as an average 20 females become victim of CA cervix daily and fall among top ten countries having highest women mortality.^14^ According to WHO; by 2030; half a million females encountered deaths from which 98% deaths are from developing countries like Pakistan.^19^ The barrier against awareness and screening is the abysmal health promotion and disease prevention framework of our country. Call for awareness drive is the powerful tool which can change the mindset of the Pakistani inhabitants to accept and understand the importance of prevention is better than cure.

**Table-III:** Binary Logistic regression of influence of awareness and counseling over cases and controls.

Variables	B	S.E	Wald	df	Sig	Exp B	95% C.I. for EXP (B)	

							Lower	Upper
Family history of CA Cervix	1.554	.342	20.634	1	0.000	4.728	2.38	7.53
Knowledge of PAP-Smear Test!	0.079	.506	0.024	1	0.045	1.082	0.97	5.36
Ever counseled for PAP-Smear.	-1.484	.446	11.061	1	0.001	0.227	0.2	0.79
Ever been tested for PAP-Smear	-1.150	.798	2.076	1	0.150	0.317	0.13	1.84
Due to cost; never been tested for PAP-smear.	0.915	0.417	4.816	1	0.028	2.496	0.92	3.3
Constant	0.405	0.129	9.864	1	0.002	1.500		

Variable(s) entered on step 1: Family history of CA Cervix, importance of PAP smear, ever counseled for PAP smear, ever tested for PAP smear, never been tested for PAP smear due to cost.

According to the study of Khan et al;^20^ only 4.3% females were vaccinated against HPV, 70% women disregarded this malignancy due to timid female nature and discouraged family circumstances and more over majority of health professionals were unaware of the correct position of CA cervix.^21^

In this catastrophe; primary healthcare provider has a significant role to play proficiently for examination, increase awareness level, and counseling of PAP smear^22^ to battle against CA cervix. Family history of CA Cervix, importance of PAP smear, ever counseled for PAP smear, ever tested for PAP smear, never been tested for PAP smear due to cost were significantly predicted factors for CA cervix among controls in this study. Total 21.6% participants were counseled by primary healthcare providers for risk factors of CA cervix and 9.6% had the knowledge of PAP smear screening in our study. Hence; healthcare providers are abysmal healthcare promoter in our case it might be due to socio demographic covariates in rural and urban areas in this study. The knowledge was better than the study of Minhas et al^13^, this study revealed 3% knowledge about cervical cancer screening among females of Punjab, Pakistan. On the contrary Ozcan H et al;^23^ revealed that 48.6% Turkstat had knowledge of screening of CA cervix and 16.2% received this knowledge from healthcare provider. Regrettably; PAP smear testing has not been implemented properly in Pakistan. There is a long way to go for routine practice of vaccination and PAP smear testing in our country which is possible through structured healthcare system.

### Limitations of the Study:

Despite being a case control study and adequate sample size; there are some limitations which must be addressed. Due to the cultural values and conservative norms; it was very difficult to interrogate for multiple sexual partners thus have to tackle the situation very tactfully. Though; Pakistan has Muslim culture and safe sex is practiced. But in some society’s moral and social dilemmas exists having multiple sex partners as risk factor for CA cervix.^13^ Proper counseling by primary healthcare provider can increase the awareness of risk factors and prevention of CA cervix among females.

Cost of PAP smear is one of the reasons for not tested for CA cervix in this study. Hence; this is recommended that PAP smear screening and HPV vaccination should be cost effective, accessible and affordable test in our healthcare system. Primary healthcare providers should be trained in such a way as to strengthen awareness, healthy life style, behavioral change communication^18^ without discrimination of having any educational status, urban and rural habitants; as mothers are agents of change to bring their daughters for vaccinations before sexual activity and regular PAP smear screening.

## CONCLUSION

It was inferred from the study that educational level, age at first intercourse, number of parity was the risk factors of CA cervix. Family history of CA Cervix, knowledge of cervical cancer screening, ever counseled for PAP smear, ever tested for PAP smear, never been tested for PAP smear due to cost were significantly predicted for CA cervix among controls.

### Authors’ Contribution:

**HS:** Project concept & study design, Data collection, literature review, take the overall responsibility & accountable for the accuracy or integrity of the work.

**IMH:** Data collection reviewed and edited the manuscript.

**KFMB**: Manuscript write up, Statistical analysis and interpretation of results, formal analysis and final approval of the manuscript.
